# Increased cerebral expressions of *MMPs*, *CLDN5*, *OCLN*, *ZO1* and *AQPs* are associated with brain edema following fatal heat stroke

**DOI:** 10.1038/s41598-017-01923-w

**Published:** 2017-05-10

**Authors:** Yu Du, Jing-Tao Xu, Hong-Nian Jin, Rui Zhao, Dong Zhao, Si-Hao Du, Ye Xue, Xiao-Li Xie, Qi Wang

**Affiliations:** 1 Department of Forensic Medicine, National Police University of China, Shenyang, China; 2 Department of Forensic Pathology, School of Forensic Medicine, Southern Medical University, Guangzhou, China; 3 Forensic Science Centre of Guangdong Provincial Public Security Department, Guangzhou, China; 4 Department of Forensic Pathology, China Medical University School of Forensic Medicine, Shenyang, China; 5 Collaborative Innovation Center of Judicial Civilization, China; Key Laboratory of Evidence Science (China University of Political Science and Law), Ministry of Education, Beijing, China; 6 Department of Toxicology, School of Public Health, Southern Medical University, Guangdong Provincial Key Laboratory of Tropical Disease Research, Guangzhou, China

## Abstract

Human brain samples were collected from 46 autopsy cases, including 23 fatal heat stroke cases and 23 age-matched controls. Nine candidate reference genes (*PES1*, *POLR2A*, *IPO8*, *HMBS*, *SDHA*, *GAPDH*, *UBC*, *B2M*, *ACTB*) were evaluated in the cerebral cortex of 10 forensic autopsy cases (5 heat stroke and 5 controls), using the geNorm module in qBase^plus^ software. *SDHA*, *POLR2A*, *IPO8* and *HMBS* were identified as the most stable reference genes. Using these validated reference genes, mRNA expressions of *Matrix metalloproteinases* (*MMPs*, *MMP2* and *MMP9*), *Claudin5* (*CLDN5*), *Occludin* (*OCLN*), *Zona occludens protein-1* (*ZO1*) and *Aquaporins* (*AQPs*, *AQP1* and *AQP4*) in the cerebral cortex were examined. Relative mRNA quantification using Taqman real-time PCR assay demonstrated increased calibrated normalized relative quantity (CNRQ) values of *MMP9*, *CLDN5*, *OCLN*, *ZO1* and *AQP4* in heat stroke cases. Heat stroke cases showed an increase in brain water content, which was found to be positively correlated with *MMP9*, *OCLN*, *ZO1* and *CLDN5* mRNA. When using one conventional reference gene (*GAPDH* or *ACTB*) for normalization, no difference was detected between heat stroke and controls. In immunostaining, only *AQP4* showed more intense staining in most heat stroke cases. The present study, for the first time, reports increased cerebral *MMP9*, *CLDN5*, *OCLN*, *ZO1* and *AQP4* in heat stroke and suggest a crucial role of reference gene selection when using postmortem human tissues.

## Introduction

Heat stroke is defined as a form of hyperthermia associated with a systemic inflammatory response leading to a syndrome of multi organ dysfunction in which encephalopathy predominates^1^. The mortality is as high as 10–15% in patients with heat stroke. Nearly 30% of patients with heat stroke are accompanied by central nervous system (CNS) dysfunction that results in delirium, convulsions, or coma^2^. Brain edema is an important factor associated with brain damage causing long-term disability and death in patients with heat-related illness. In fact, forensic autopsy data showed profound brain edema in heat stroke cases^3^. However, this phenomenon has not been fully emphasized in clinical treatment. The potential mechanism of brain edema formation following heat stroke has not been fully clarified.

*Matrix metalloproteinases* (*MMPs*) belong to a family of calcium-dependent zinc-containing endopeptidases, which are involved in the tissue remodeling and degradation of the extracellular matrix (ECM)^4^. Considerable research has been conducted on the role of two secreted *MMPs*, *MMP2*, and *MMP9*. Both of them have positive and negative roles in the healthy and diseased CNS^5^. *MMP9* is responsible for blood-brain barrier (BBB) opening in several pathological conditions and the marked increase of *MMP9* causes severe BBB disruption. *MMP9*-mediated BBB disruption may be a crucial step in the pathogenesis of some neuroinflammatory diseases^6–8^. *Claudin5* (*CLDN5*), *Occludin* (*OCLN*) and *Zona occludens protein-1* (*ZO1*) are key tight junction (TJ) proteins that play an important role in modulation of BBB permeability^9^. Decreased expression of *CLDN5*, *OCLN* and *ZO1* has been reported to be closely associated with BBB damage^10^. *Aquaporins* (*AQPs*) are water channels that facilitate water transport. *AQP1* and *AQP4* are presumed as major contributors to participate in brain water homeostasis^11^. *AQPs*, in particular *AQP4*, appear to play a crucial role in cerebral volume regulation following trauma, inflammation, ischemia, tumors as well as metabolic disturbances^12, 13^.

Experimental studies reported that hyperthermia caused by heat exposure is closely associated with the breakdown of the BBB followed by brain edema^14^. The disruption of the BBB is characterized by the degradation of the junctional complex proteins and increase in multiple *matrix metalloproteinases*. However, these studies have generally been restricted to rodent models that have inevitably inherent deficiencies, including a lissencephalic brain and small head size relative to body size. Furthermore, heat stroke in human is rarely as pure as in experimental models, it is necessary to investigate the human materials following fatal heat stroke.

The present study analyzed the gene expressions of *MMP2*, *MMP9*, *CLDN5*, *OCLN*, *ZO1*, *AQP1* and *AQP4*, in the brains of forensic autopsy cases, using reverse transcription quantitative PCR (RT-qPCR), combined with immunohistochemical detections, to investigate the molecular pathology of brain edema in fatal heat stroke cases with special regard to the importance of reference gene selection.

## Results

### Brain water content

There was a significant increase in brain water content in the heat stroke group as compared to the control group (Table 1, heat stroke: 82.3% ± 2.9%; control: 79.3% ± 2.6%, p < 0.05, Student’s t test).

**Table 1:** Case profiles n = 46.

Cause of death	n	M/F	Age (years) mean ± SD	PMI (h) mean ± SD	RIN (mean ± SD)	Brain water content, % mean ± SD
Heat stroke	23	13/10	58.5 ± 9.5	26.0 ± 8.1	3.6 ± 1.0*	82.3 ± 2.9*
control	23	13/10	56.6 ± 8.8	27.3 ± 7.8	4.9 ± 1.1	79.3 ± 2.6
Total	46	26/20	57.5 ± 9.1	26.6 ± 7.9	4.2 ± 1.2	80.8 ± 3.1

Significantly lower RIN values were detected in heat stroke group as compared to the control group (*p < 0.05).

There was a significant increase in brain water content in the heat stroke group as compared to the control group (*p < 0.05).

### RNA concentration, purity and integrity

RNA concentrations ranged from 25.9 to 348.7 ng/μL (mean 194.1 ng/μL). There were no age or postmortem interval dependences on Pearson correlation analysis (p > 0.05). RNA concentrations showed no significant differences between the heat stroke group and control group.

RNA purity, determined using 260/280 absorbance (A_260_/A_280_) ratios, ranged from 1.75 to 2.19 (mean 1.98). There were no age or postmortem interval dependences on Pearson correlation analysis (p > 0.05). RNA purity showed no significant differences between the heat stroke group and the control group.

RIN values showed substantial variations in each group. There were no age or postmortem interval dependences on Pearson correlation (p > 0.05). RIN values were evidently lower in the heat stroke group as compared to the control group (Table 1, heat stroke: 3.6 ± 1.0; control: 4.9 ± 1.1, p < 0.05, Student’s t test).

### Amplification efficiency

The amplification efficiencies of targets and reference genes ranged from 86.2% (*ACTB*) to 104.6% (*UBC*), showing small inter-individual variations (standard deviation, SD < 5%). Details are shown in Table 2.

**Table 2:** Introduction of the PCR primers and probes.

Gene Symbol	Gene Name	TaqMan Assay ID	Amplicon Length (bp)	Amplification efficiency (mean ± SD)
**Gene of interest**				
*AQP1*	*aquaporin 1*	Hs00166067_m1	86	0.896 ± 0.021
*AQP4*	*aquaporin 4*	Hs00242342_m1	92	0.955 ± 0.014
*CLDN5*	*claudin 5*	Hs01561351_m1	55	0.903 ± 0.016
*MMP2*	*matrix metallopeptidase 2*	Hs01548727_m1	65	0.953 ± 0.031
*MMP9*	*matrix metallopeptidase 9*	Hs00234579_m1	54	0.939 ± 0.028
*OCLN*	*occludin*	Hs00170162_m1	68	1.0387 ± 0.039
*ZO1*	*zona occludens protein-1*	Hs01551861_m1	148	0.974 ± 0.028
**Reference gene**				
*PES1*	*pescadillo homolog 1*	Hs00362795_g1	56	0.985 ± 0.029
*POLR2A*	*polymerase* (*RNA*) *II* (*DNA directed*) *polypeptide A*	Hs00172187_m1	54	1.010 ± 0.017
*IPO8*	*importin 8*	Hs00183533_m1	71	1.025 ± 0.033
*HMBS*	*hydroxymethylbilane synthase*	Hs00609297_m1	64	1.019 ± 0.025
*SDHA*	*succinate dehydrogenase complex*	Hs00188166_m1	70	1.018 ± 0.037
*GAPDH*	*glyceraldehyde-3-phosphate dehydrogenase*	Hs99999905_m1	122	0.927 ± 0.016
*UBC*	*ubiquitin C*	Hs00824723_m1	71	1.046 ± 0.027
*B2M*	*beta-2-microglobulin*	Hs99999907_m1	75	1.041 ± 0.037
*ACTB*	*beta-actin*	Hs99999903_m1	171	0.862 ± 0.019

“_m” indicates the assay’s probe spans an exon junction and will not detect genomic DNA. “_g” indicates the assay may detect genomic DNA. Detailed information for each TaqMan Assay is available from Applied Biosystems.

### Reference genes validation

**Figure 1:**
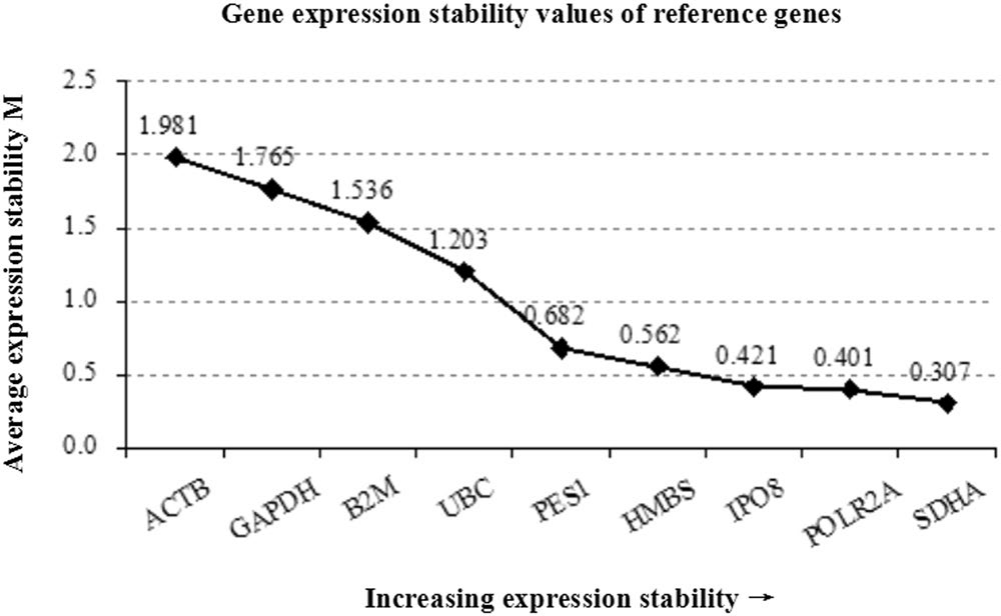
Average expression stability values (M). Expression stability values of genes from the least stable (left) to most stable (right).

**Figure 2:**
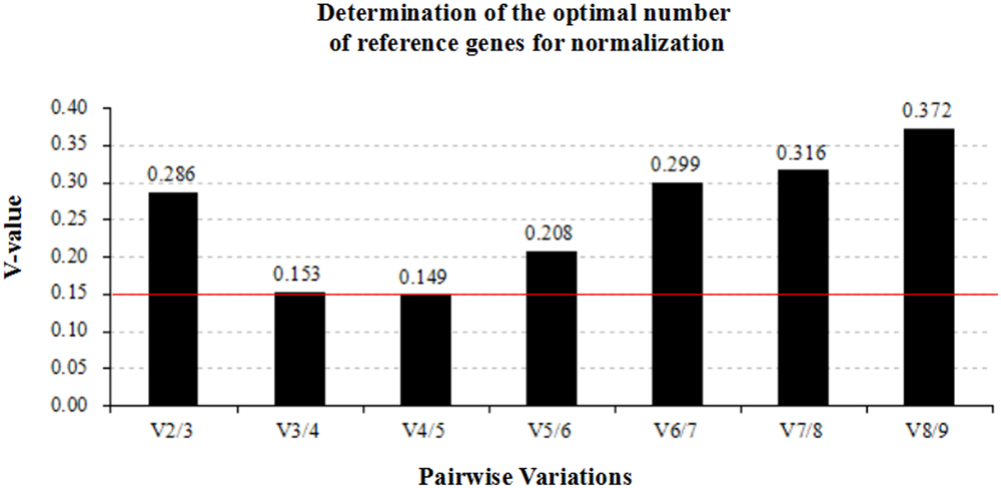
Pairwise variation of candidate reference genes using geNorm analysis. Pairwise variation analysis to determine the optimal number of reference genes for normalization.

The geNorm module in qBase^plus^ software ranked the 9 reference genes. The most stable one was *SDHA* followed by *POLR2A*, *IPO8* and *HMBS*. The least stable one was *ACTB* (Fig. 1). Pairwise variation (V) was calculated based on normalization factor values (NFn and NFn + 1) after the inclusion of the least stable reference gene and indicated if the extra reference gene added to the stability of the normalization factor. The V-value was the lowest when the fifth most stable gene (*PES1*) was added (Fig. 2). However, when the fourth most stable gene (*HMBS*) was added, V3/4 showed a V-value of 0.153, near the threshold of 0.15. Therefore, to save on cost and time, four reference genes *SDHA*, *POLR2A*, *IPO8* and *HMBS*, were selected for normalization.

## Data analysis

### Normalization against validated reference genes

Raw Ct values and amplification efficiencies of targets and 4 validated reference genes, *SDHA*, *POLR2A*, *IPO8* and *HMBS*, were imported into the qBase^plus^ software. CNRQ values were exported and statistically investigated.

There were no gender-related differences, or age or postmortem interval dependence in CNRQ values of target genes on Pearson correlation (p > 0.05).

**Figure 3:**
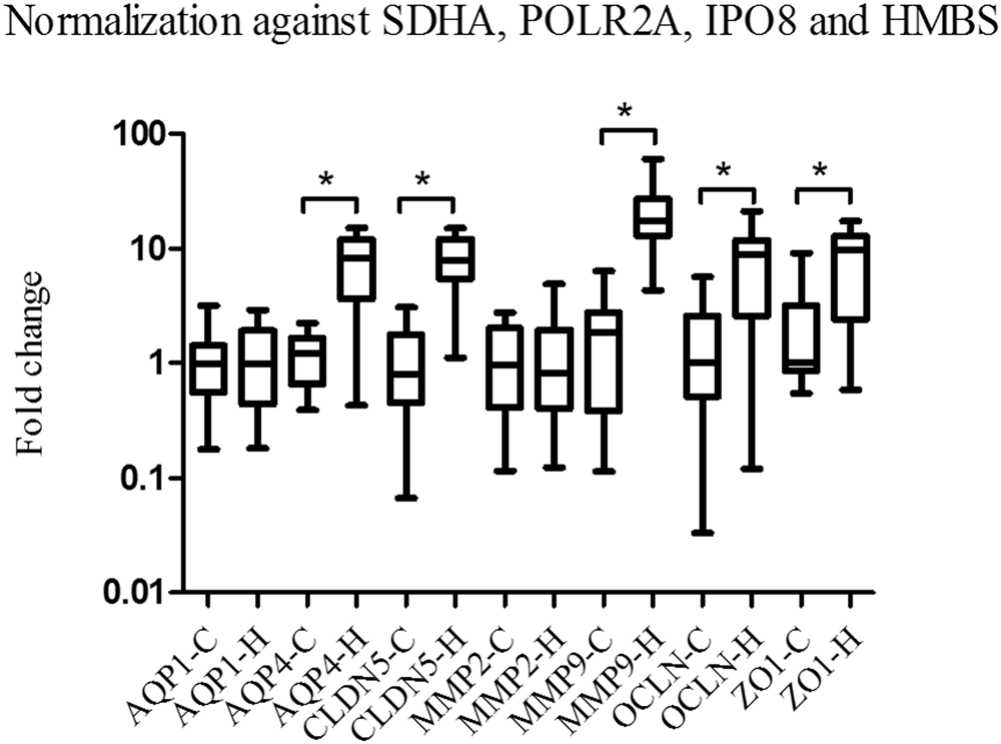
*AQP1*, *AQP4*, *CLDN5*, *OCLN*, *ZO1*, *MMP2* and *MMP9* mRNA expression levels after normalization against four validated reference genes. CNRQ values of *AQP4*, *CLDN5*, *OCLN*, *ZO1* and *MMP9* were significantly higher in the heat stroke group as compared to the control group.

CNRQ values of *AQP4*, *CLDN5*, *OCLN*, *ZO1* and *MMP9* were significantly higher in the heat stroke group as compared to the control group (Fig. 3).

CNRQ values of *CLDN5* and *MMP9* were found to be positively correlated with brain water contents (r^2^ = 0.1225 and 0.1486, p < 0.05).

### Normalization against *ACTB* or *GAPDH*

**Figure 4:**
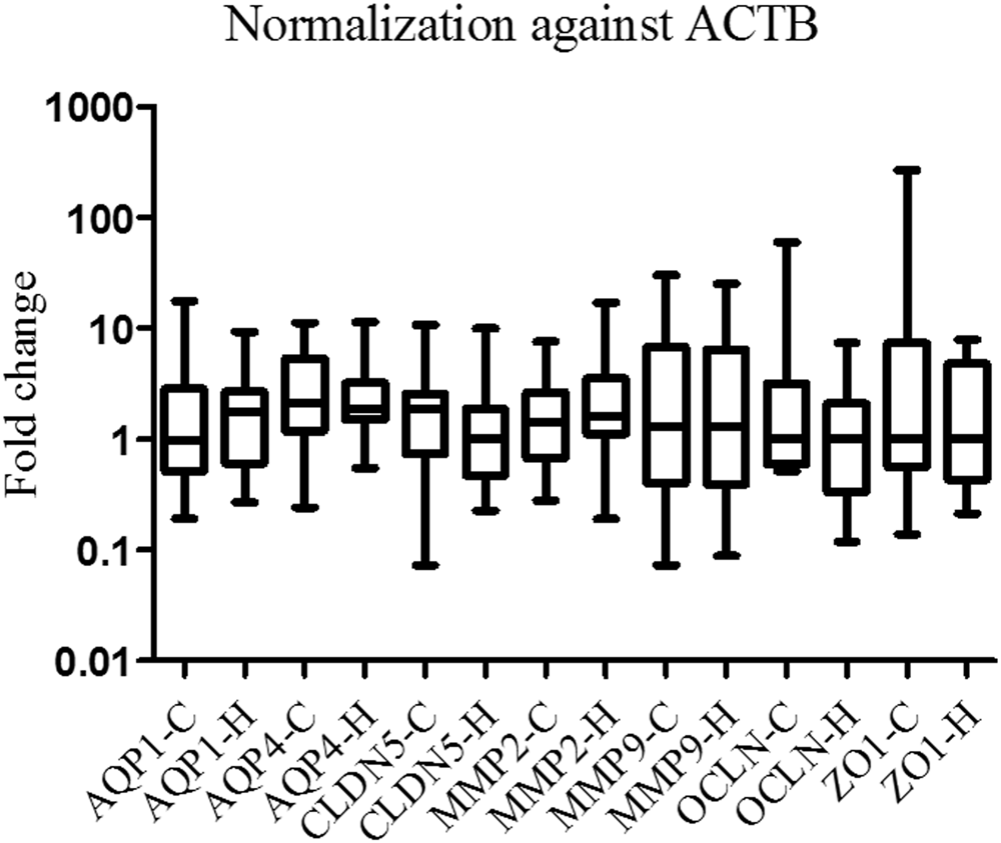
*AQP1*, *AQP4*, *CLDN5*, *OCLN*, *ZO1*, *MMP2* and *MMP9* mRNA expression levels after normalization against *ACTB*. There was no significant difference in the expression of any target gene.

**Figure 5:**
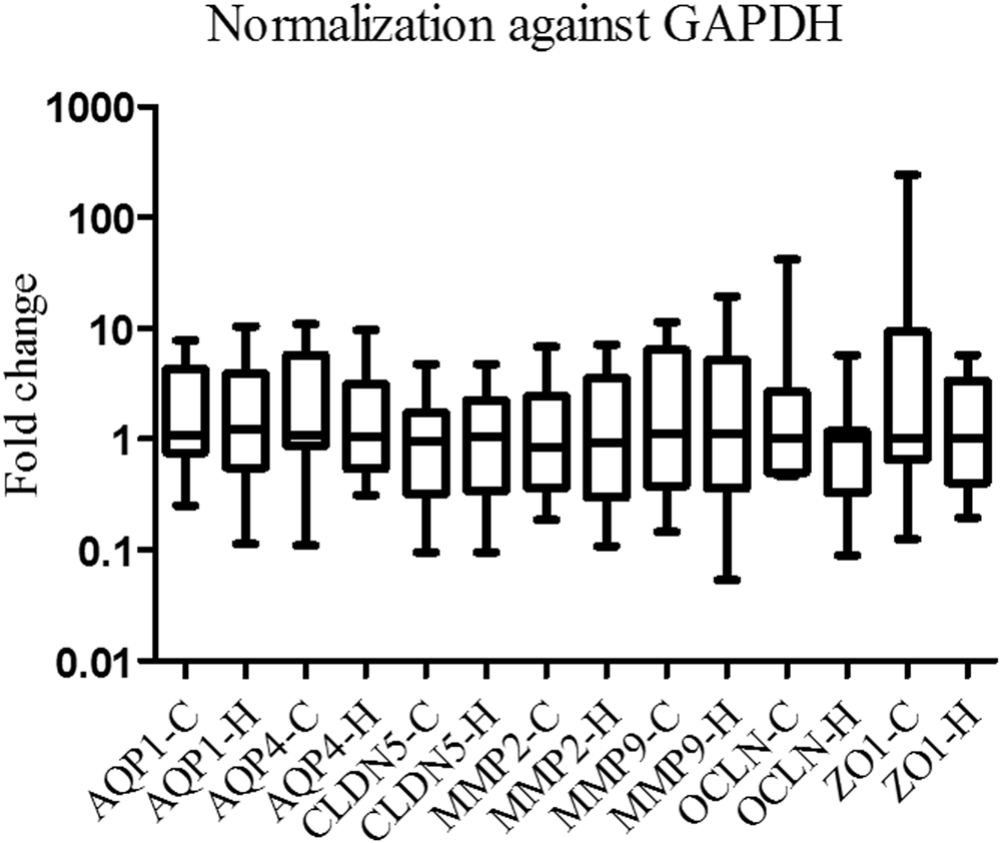
*AQP1*, *AQP4*, *CLDN5*, *OCLN*, *ZO1*, *MMP2* and *MMP9* mRNA expression levels after normalization against *GAPDH*. There was no significant difference in the expression of any target gene.

When *ACTB* or *GAPDH* alone was used for normalization, there was no significant difference in the expression of any target gene (Figs 4 and 5).

#### Immunostaining

**Figure 6:**
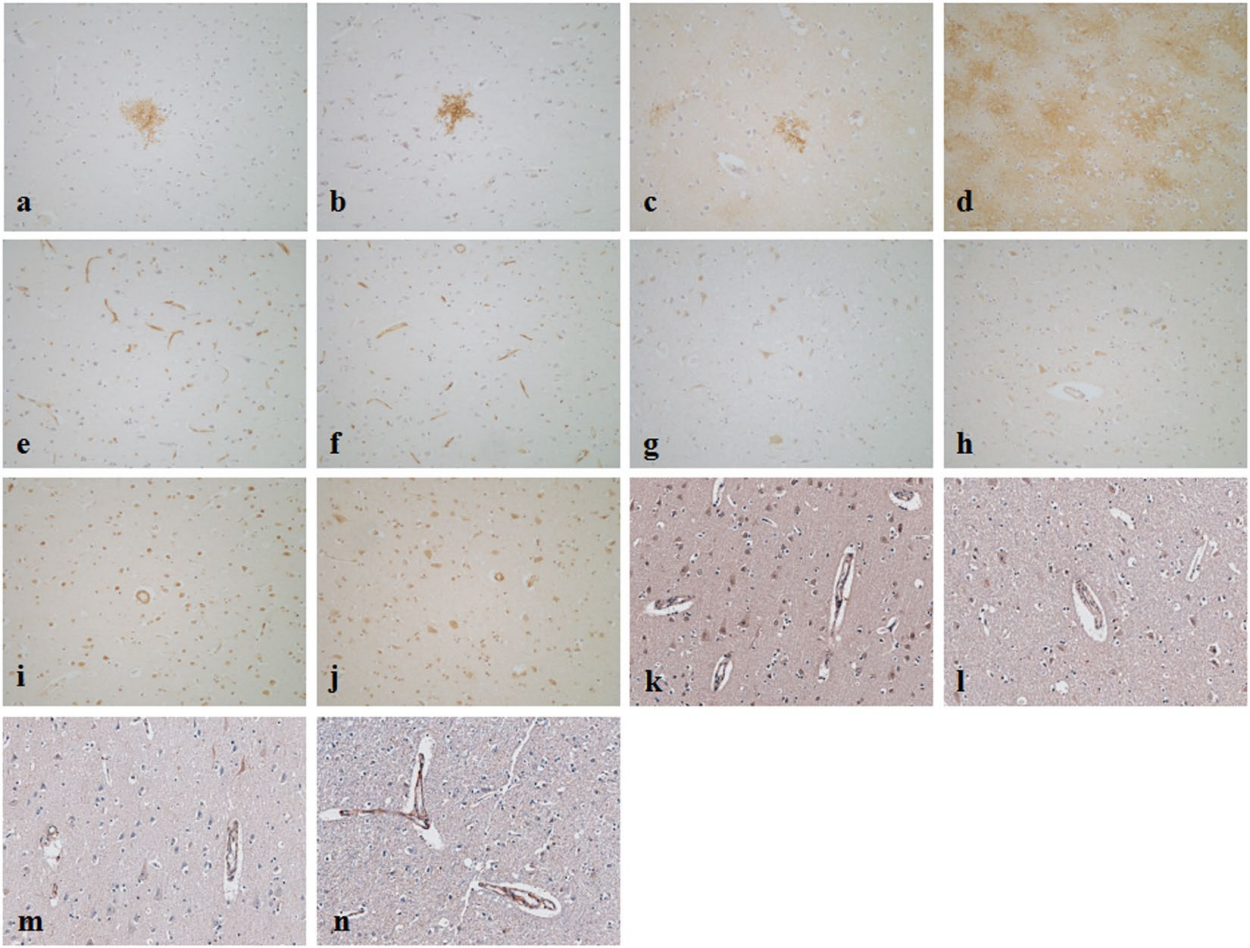
Immunostaining of *AQP1* (**a** and **b**), *AQP4* (**c** and **d**), *CLDN5* (**e** and **f**), *MMP2* (**g** and **h**), *MMP9* (**i** and **j**), OCLN (**k** and **l**) and ZO1 (**m** and **n**) in the brain. Peracute death due to blunt injury (**a**,**c**,**e**,**g**,**i**,**k** and **m**), a 52-year-old male, 27 h postmortem. Death due to heat stroke (**b**,**d**,**f**,**h**,**j**,**l** and **n**), a 64-year-old male, 30 h postmortem.

**Figure 7:**
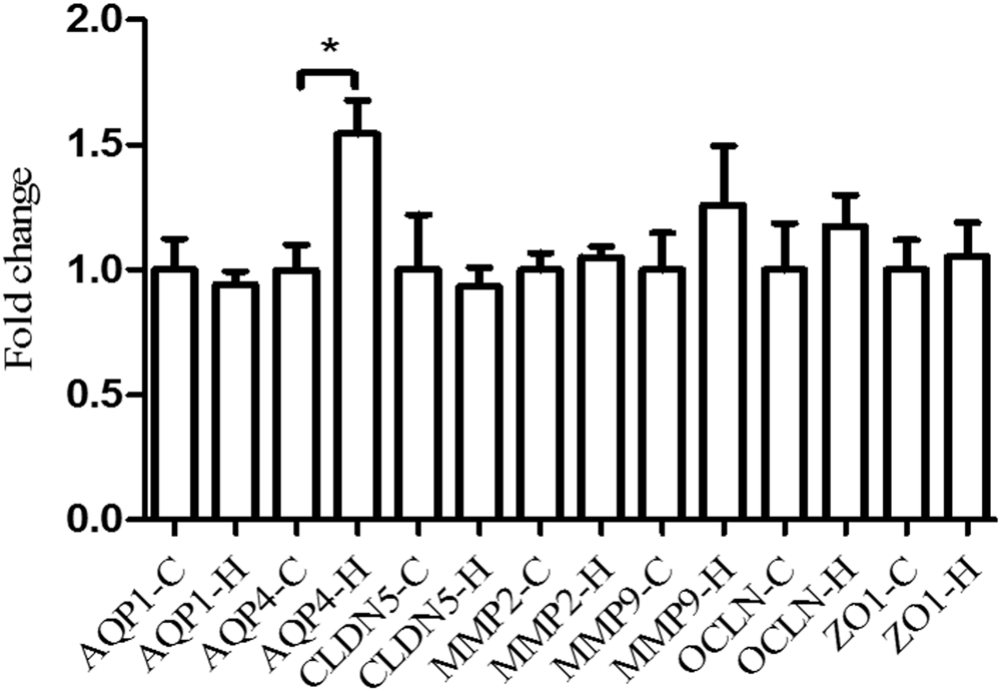
Quantification of immunostaining data. Ratio for fold change of *AQP4* was significantly higher in the heat stroke group as compared to the control group. Significantly lower RIN values were detected in heat stroke group as compared to the control group (*p < 0.05).

Immunostaining showed substantial interindividual variations in each group. *AQP1* (Fig. 6a and b) and *AQP4* (Fig. 6c and d, Fig. 7) were mainly detected in glial cells which were morphologically identified as astrocytes, and only *AQP4* showed more intense staining in most heat stroke cases. *CLDN5* was strongly positive in capillary endothelia, and no significant differences in distribution or intensity were detected between heat stroke and control group (Fig. 6e and f, Fig. 7). *MMP2* was detected clearly in the neurons, showing no significant differences in distribution or intensity between heat stroke and control group (Fig. 6g and h, Fig. 7). *MMP9* was located in glial cells, neurons and capillary endothelia, and no significant differences in distribution or intensity were detected between heat stroke and control group (Fig. 6i and j, Fig. 7). *OCLN* was positive in capillary endothelia, sporadically in neurons and glial cells, and no significant differences in distribution or intensity were detected between heat stroke and control group (Fig. 6k and l, Fig. 7). *ZO1* was located in capillary endothelia, and no significant differences in distribution or intensity were detected between heat stroke and control group (Fig. 6m and n, Fig. 7).

## Discussion

RT-qPCR is increasingly applied to determine changes in gene expressions due to the high sensitivity and accuracy of the technique. The most common procedures in RT-qPCR are relative measurements of genes of interest after normalization with the endogenous reference gene(s). Accurate and reliable relative RT-qPCR requires ideal reference gene(s). However, expressions of several conventional reference genes were shown to vary due to nutritional or hormonal factors, biological processes, and/or tissue or cell types; a single endogenous reference gene cannot meet the criteria of an ideal reference gene^15^. In the field of molecular neurobiology, RT-qPCR, using postmortem autopsy tissues, has become a hotspot^16–18^. It can provide novel biomarkers and disease-modifying therapeutic targets for some CNS diseases. Previous studies, using different normalization strategies, demonstrated contradicting results, suggesting the importance of normalization^19, 20^.

In the present study, with the help of geNorm module in qBaseplus software, nine reference genes were evaluated. The V-value was the lowest when the fifth most stable gene (*PES1*) was added. Further addition of genes increased V-values, indicating a negative influence on the normalization process. Ideally, a threshold V-value of 0.15 is recommended as a cut-off value by geNorm to determine the optimal number of reference genes^21^. However, when the fourth most stable gene (*HMBS*) was added, V3/4 showed a V-value of 0.153, near the threshold of 0.15. Therefore, to save on cost and time, four reference genes *SDHA*, *POLR2A*, *IPO8* and *HMBS*, were selected for normalization. In the present study, CLW/H ratios were higher in the heat stroke group as compared to the control group, indicating brain edema in heat stroke cases. However, using mRNA measurements of intracerebral *MMPs*, *CLDN5*, *OCLN*, *ZO1*, and *AQPs* as markers of brain edema, inconsistent results were detected by different normalization methods. When those four validated reference genes, *SDHA*, *POLR2A*, *IPO8* and *HMBS*, were used for normalization, increased cerebral expressions of *AQP4*, *CLDN5*, *OCLN*, *ZO1* and *MMP9* were detected in heat stroke group. However, these findings cannot be detected when *GAPDH* or *B2M* alone was used for normalization. Expression stability values of these five reference genes calculated by geNorm showed *ACTB* as the least stable one, followed by *GAPDH*. Therefore, gene expression levels that normalized against four validated reference genes were believed to be accurate and reliable; *ACTB* and *GAPDH*, two conventional reference genes, were not suitable for normalization of human postmortem brain tissues.

Another considerable factor influencing the accuracy of gene expression analysis using RT-qPCR is the integrity of RNA^22^. However, unlike animal experimentation, RNA degradation is inevitable and unpredictable for human tissues collected at autopsy. In the present study of the human brain tissues, RIN values showed no postmortem interval-dependent changes but were significantly lower in the heat stroke group as compared to the control group, indicating that RNA quality was more seriously affected in cases of hyperthermia.

The up-regulations of *MMP2* and *MMP9* in brain are associated with an increase of BBB permeability by degrading the endothelial basal lamina of the BBB which results in vasogenic edema^23^. Despite the well documented effects of systemic inflammatory response, the impact of hyperthermia on the BBB has been overlooked and the probable mechanism has not been fully addressed. In the present study, brain tissues in heat stroke group showed evidently higher CNRQ values of *MMP9*, but not *MMP2*. These findings suggest independent contributions of *MMP2* and *MMP9* in the brain tissues of heat stroke group, which require further investigation. *MMP9* was regarded as a key player in the alteration of BBB permeability. Several studies in animal models have shown that increased *MMP9* is closely related to the breakdown of BBB, by digesting ECM^24, 25^. Both inhibiting *MMP9* and deleting *MMP9* gene can attenuate the BBB disruption^26, 27^.

In addition, intercellular junctions between endothelial cells are essential for vascular integrity and function. Breaching of endothelial barriers is a key event in the development of brain edema. The loss of *CLDN5*, *OCLN* and *ZO1* in TJs can open the BBB, lead to brain edema and neuronal cell death. Decreased expression of *CLDN5*, *OCLN* and *ZO1* is closely associated with BBB damage^28^. However, in the present study, increased *CLDN5*, *OCLN* and *ZO1* mRNA expression was detected in the heat stroke group. These findings might be considered a compensatory mechanism to mend junctional complexes and restore barrier function.

*AQP4*, the principal *AQP* in mammalian brain, highly correlates with a variety of pathophysiological processes of brain edema. Increased expression of *AQP4* in the brain indicated that *AQPs* participated in the formation of brain edema^29^. In the vasogenic edema resolution phase, an increase of *AQP4* was observed in several studies^30, 31^. Therefore, in heat stroke cases, *AQP4* might play a beneficial role in eliminating accumulating water from the extracellular space of the CNS, suggesting an activation of the self-protective system.

In the present study, the immunostaining did not detect any evident differences in distribution or intensity among all the causes of death except for *AQP4*. These findings may be caused by a lower sensitivity of immunostaining in detecting changes in gene products than that with quantitative analyses of gene expressions using RT-qPCR. The major limitation of the present study is that the protein levels of all targets have not been examined because of the limitation of the postmortem materials, which need further investigation.

In conclusion, the present study shows that brain edema is evident in heat stroke, and human brain retains a self-protective response capacity in victims who died due to heat stroke. Systematic analysis of gene expressions using RT-qPCR is a useful procedure and validation of reference genes is crucial.

## Materials and Methods

### Sample collection

A total of 46 human forensic autopsy cases were selected from autopsy documents. The demographics of study subjects are described in Table 1. In each case, the cause of death was carefully diagnosed on the basis of autopsy examination, including macromorphological, histological, toxicological and biochemical analyses. Cases were divided into two groups as follows: 23 fatal heat stroke cases and 23 age, gender and postmortem interval (PMI)-matched control subjects, including three hanging cases, three strangulation cases, five blunt injury cases, six fire fatality cases, and six acute cardiac death cases. A thorough neuropathological analysis was performed as part of our routine investigation, and cases with any preexisting neurological pathologies were excluded in the present study.

In the present study, clearly accountable cases without any other complications that may have contributed to the death, supported by well-established circumstantial evidence, were collected. Postmortem interval was defined as the estimated time from death to autopsy which was estimated on the basis of autopsy findings and circumstantial evidence recorded in autopsy documents. The definition and criteria of deaths due to heat stroke are described in our previous report^32^; drug abusers and chronic alcoholics were excluded from the heat stroke group. This work was approved by our institutional Ethics Committee of Southern Medical University. All sampling methods were followed anatomical practices and carried out in accordance with regulations of *Methods of extraction*, *fixation*, *packing and inspection of forensic pathology of The PRC Public Safety Industry Standard* (*GA/T 148–1996*) and *Forensic pathology materials extraction*, *fixed operating instructions of Southern Medical University* (*NYSJ-JS-BL04*). As one of our routine work, the informed consent paper was obtained from the immediate family members of deceased before starting autopsy.

### Brain water content

Brain water content was measured by a halogen moisture analyzer (model HB43, METTLER TOLEDO, Switzerland) automatically according to the manufacturer’s instructions^20^. Briefly, brain tissue samples were taken from consistent sites in the parietal lobe of left cerebral hemispheres at autopsy. About one gram of brain tissue was weighted first to obtain a wet weight (WW), then placed in the analyzer at 150 °C for about 30 min and weighted again to obtain a dry weight (DW). The water content was calculated using the equation: (WW − DW)/WW × 100%.

### Toxicological analyses

The procedures of drug testing and analysis, including chemicals and reagents, sample preparation and conditions of the instrument were performed by gas chromatography/mass spectrometry^33^.

### Extraction of total RNA and cDNA synthesis

Tissue specimens were taken from consistent sites in the central anterior of left cerebral hemispheres (precentral gyrus) at autopsy, then immediately submerged in 1 ml of RNA stabilization solution (RNAlaterTM, Ambion, Austin). Total RNA was isolated from 100 mg of sample using RNAiso Plus (Takara Bio, Inc., Shiga, Japan) according to the manufacturer’s instructions. After extraction, the RNA concentration was estimated by spectrophotometric analysis using a NanoDrop 1000 (Thermo Scientific, Wilmington, USA). cDNA copies of total RNA were obtained using a High Capacity RNA-to-cDNA kit (Applied Biosystems Japan, Ltd.), then were adjusted to a concentration equivalent to 5 ng/μl of total RNA using nuclease-free water.

### Evaluation of the quality and integrity of RNA samples

RNA purity was determined using 260/280 absorbance (A_260_/A_280_) ratios. The RNA integrity number (RIN) was determined using a RNA 6000 Nano Labchip kit in an Agilent 2100 Bioanalyzer (Agilent Technologies, Palo Alto, USA) following the manufacturer’s protocol.

### Reference genes selection

Nine candidate reference genes were evaluated in the brain tissue of 10 forensic autopsy cases (Heat stroke, n = 5 and control, n = 5): *pescadillo homolog 1* (*PES1*), *polymerase* (*RNA*) *II* (*DNA directed*) *polypeptide A* (*POLR2A*), *importin 8* (*IPO8*), *hydroxymethylbilane synthase* (*HMBS*), *succinate dehydrogenase complex* (*SDHA*), *glyceraldehyde-3-phosphate dehydrogenase* (*GAPDH*), *ubiquitin C* (*UBC*), *beta-2-microglobulin* (*B2M*), *beta-actin* (*ACTB*). *PES1*, *POLR2A*, *IPO8*, *HMBS*, *SDHA* were chosen from the relevant literature and have been validated in postmortem brain tissues^34, 35^. *GAPDH*, *B2M* and *ACTB* were conventional reference genes. Details are shown in Table 2.

### Quantitative real-time reverse transcriptase polymerase chain reaction (RT-qPCR)

The PCR primers and probes (TaqMan Gene Expression Assay) were purchased from Applied Biosystems, Inc. (Carlsbad, CA, USA). Details are shown in Table 2. RT-qPCR reactions were run in 96-well reaction plates with an Applied Biosystems 7500 Fast Real-Time PCR system (Applied Biosystems, Foster City, CA, USA). RT-qPCR was performed with 10 μl cDNA (corresponding to the cDNA reverse transcribed from approximately 50ng RNA) in 20 μl reaction mix containing 10 μl TaqMan Gene Expression Master Mix (2×) and the above-mentioned TaqMan Gene Expression Assays (lyophilized powder). Thermal cycling conditions included 1 cycle at 50 °C for 2 min, 1 cycle at 95 °C for 10 min, followed by 40 cycles of amplification at 95 °C for 15 sec, and 60 °C for 1 min. The threshold cycle (Ct) was calculated by the instrument software automatically (threshold value at 0.2). Raw fluorescent data (normalized reporter values, Rn values) were also exported.

### Amplification efficiency calculation

Amplification efficiencies were calculated from raw fluorescent data (Rn values), using a completely objective and noise-resistant algorithm, the Real-time PCR Miner program^36^ (http://ewindup.info/miner/).

## Data analysis

### Normalization against validated reference genes

Raw Ct values and calculated amplification efficiencies of these 9 reference genes were imported into the qBase^plus^ software^37^. In qBase^plus^ software, geNorm module was used to identify the most stable reference genes and determine the minimum number of reference genes^21^.

After determining the minimum number of reference genes, raw Ct values and amplification efficiencies of targets and validated reference genes were imported into the qBaseplus software again. Using a calibrator case (peracute death due to blunt injury, 52-year-old male; 27 h postmortem), calibrated normalized relative quantity (CNRQ) values were exported from the qBaseplus software, and statistically investigated.

### Normalization against conventional reference gene (*ACTB* or *GAPDH*)

The expression levels of mRNA transcripts are described as the ratios of the targets normalized to the endogenous reference (*GAPDH* or *B2M*), using the 2^−ΔΔCt^ method, as the ratios for fold change relative to the above mentioned calibrator. The 2^−ΔΔCt^ method assumes that the amplification efficiency of the reaction is ideal (100%) and constant for each sample.

### Immunostaining

The brains were fixed in buffered 4% formaldehyde for 2 weeks and the cerebral hemispheres were cut coronally into 1-cm-thick sections, and the sections of cerebellum and brain stem were also prepared. Paraffin-embedded brain tissue specimens were taken from the standardized anatomical regions. Serial sections (5 μm thick) were cut and stained with hematoxylin-eosin (HE) as part of routine laboratory investigation. In the present study, parietal lobes of left cerebral hemispheres were used for immunostaining.

Mouse monoclonal anti-*AQP1* antibody (Abcam, Cambridge, code ab9566, diluted 500-fold), rabbit polyclonal anti-*AQP4* antibody (Santa Cruz Biotechnology, Santa Cruz, code sc-20812, diluted 500-fold), rabbit polyclonal anti-*CLDN5* antibody (Abcam, Cambridge, code ab53765, diluted 500-fold), rabbit polyclonal anti-*MMP2* antibody (Abcam, Cambridge, code ab79781, diluted 100-fold), rabbit polyclonal anti-*OCLN* antibody (Abcam, Cambridge, code ab168986, diluted 200-fold), rabbit polyclonal anti-*ZO1* antibody (Santa Cruz Biotechnology, Santa Cruz, code sc-33725, diluted 500-fold) and rabbit polyclonal anti-*MMP9* antibody (Abcam, Cambridge, code ab38898, diluted 800-fold) were used. Following overnight incubation with the primary antibodies described above at room temperature, immunoreactions were visualized by the polymer method (ChemMate Envision, Dako, Tokyo, code k5027) and color was developed with 3,3′-diaminobenzidine tetrahydrochloride (DAB liquid system, Dako, Tokyo, code k3466), according to the manufacturer’s instructions (counterstaining with hematoxylin).

Image J. 1.46 R (NIH, USA) was used to calculate the intensity and extent of staining for the detected molecules. The per-area density of staining was calculated in this manner to reflect the percentage of the positive staining, resulting in a semi-quantitative analysis. A total of 5 microscopic fields were randomly selected, and their images were cropped. The integral optical density (IOD) levels of the stained cells in the tissue samples were then calculated by image analysis. Results were expressed as the mean ± standard deviation (SD) per tissue examined. Using a calibrator case (peracute death due to blunt injury, 52-year-old male; 27 h postmortem), ratios for fold change relative to the calibrator were used for statistical analysis.

### Statistic

All the RT-qPCR experiments were performed in triplicate, and results are reported as the mean ± SD. Correlation analyses between pairs of parameters were performed using linear regression (Pearson correlation analysis). The Student’s t test (two-tailed) was used to compare groups. Statistical analyses were performed using GraphPad Prism version 5.0 (GraphPad Software, San Diego, USA). Values of *p* < 0.05 were considered as statistically significant.
